# Increased severity and relapse rate in adult anti-nuclear matrix protein 2 antibody positive myositis patients with distal limb weakness: a retrospective study in China

**DOI:** 10.3389/fmed.2026.1873686

**Published:** 2026-06-17

**Authors:** Lin Liang, Shanshan Li, Qilu Wei, Shuo Chen, Ruilong Gao, Chao Sun, Jianyu Zhu, Jianghui Duan, Ling Zhang

**Affiliations:** 1 Department of Rheumatology, Key Laboratory of Myositis, China-Japan Friendship Hospital, Beijing, China; 2 Department of Gastroenterology, China-Japan Friendship Hospital, Beijing, China; 3 Department of Cardiology, China-Japan Friendship Hospital, Beijing, China; 4 School of Sport and Exercise Rehabilitation, Jinzhou Medical University, Jinzhou, Liaoning, China; 5 Department of Radiology, China-Japan Friendship Hospital, Beijing, China

**Keywords:** anti-nuclear matrix protein 2 antibody, distal limb weakness, multiple system involvement, myositis, relapse

## Abstract

**Purpose:**

This study aimed to examine the clinical features and prognosis of patients with distal limb weakness in anti-nuclear matrix protein 2 antibody (anti-NXP2) positive myositis.

**Patients and methods:**

This retrospective study included the medical records of patients with anti-NXP2 antibody and follow-up data. Clinical features and prognosis of patients with and without distal limb weakness were compared. The differences in groups and survival analysis were analyzed using SPSS.

**Results:**

Of 110 enrolled adult patients with anti-NXP2 positive myositis, 53 showed distal limb weakness. The frequency of distal limb weakness in patients with anti-NXP2 positive myositis was highest when compared with other dermatomyositis (DM) patients. In the patients with anti-NXP2 positive myositis, the mean onset age of distal limb weakness group was younger, when compared with those without distal limb weakness (38.40 ± 13.36 vs. 46.26 ± 15.02 years, *p* = 0.005). They exhibited higher disease activity and experienced higher frequencies of severe muscle weakness, subcutaneous edema, dysphagia, and abnormal ECG (all *p* < 0.05). In terms of treatment, 32.1% patients with distal limb weakness received glucocorticoid (GC) pulse, which was higher than those without distal limb weakness (7.0%, *p* = 0.001). There were 32 patients reporting relapse. The mean dose of GC (prednisone equivalent) at relapse was higher. Nine patients with distal limb weakness died during follow-up, with infection representing the leading cause of death. The survival rate was not different in patients with and without distal limb weakness.

**Conclusion:**

Adult patients with anti-NXP2 positive myositis and distal limb weakness were younger at disease onset and exhibited increased disease activity, including a higher prevalence of multi-system involvement, as well as a higher incidence of relapse during follow-up.

## Introduction

Proximal limb weakness is a hallmark feature of dermatomyositis (DM), and has been incorporated in both 2017 EULAR/ACR classification criteria for idiopathic inflammatory myopathies (IIMs) and 2018 European Neuromuscular Centre (ENMC) proposed criteria for DM (1, 2). However, some patients with anti-nuclear matrix protein 2 antibody (anti-NXP2) positive myositis exhibit diffuse muscle involvement, leading to clinically evident distal limb weakness (3). Moreover, a previous study observed that micro-infarction on muscle biopsy was more frequent in patients with anti-NXP2 positive myositis presenting with distal limb weakness (4). This indicates that distal weakness in patients with NXP2-positive myositis may be associated with distinct pathological features.

In addition, patients with anti-NXP2 positive myositis display substantial clinical heterogeneity (5). For instance, some individuals never develop DM specific rashes throughout the entire disease course (6). While others present prominent gastrointestinal (GI) involvement, including intestinal vasculitis and ulcers (7). Therefore, investigating the unique characteristics of patients with anti-NXP2 positive myositis complicated by distal limb weakness is of considerable clinical value.

In this study, we analyzed the clinical characteristics and prognosis of patients with distal limb weakness to provide deeper insight into anti-NXP2 positive myositis.

## Materials and methods

### Study populations

This retrospective single-center observational study enrolled adult patients with DM who were admitted to the Department of Rheumatology, China-Japan Friendship Hospital, from January 2010 to December 2023. Age at disease onset for all patients was ≥ 18 years. The diagnosis of DM met 2018 ENMC proposed criteria, and possible DM sine dermatitis (DMSD) fulfilled 2004 ENMC criteria (2, 8). There were 110 patients with anti-NXP2 antibody in the study. DM patients with other myositis specific antibody (MSA, including anti-Mi2 antibody, anti-TIF1γ antibody, anti-SAE antibody, anti-MDA5 antibody, and MSA negative) were recruited as disease control group. The study protocol was approved by the Ethics Committee of China-Japan Friendship Hospital (approval number 2016-117). Informed consents were obtained from all recruited patients.

### Clinical features of anti-NXP2 positive myositis

We collected all of the patient demographics, clinical features, and laboratory data by medical record in our department from the first hospitalization.

The main clinical features contained myalgia, muscle weakness, cutaneous involvement, cardiac involvement, GI involvement, arthritis, and interstitial lung disease (ILD). Distal limb weakness was recorded when self-reported by the patient and subsequently confirmed during clinical evaluation and physical examination performed by experienced rheumatologists. Severe muscle weakness was defined as difficulty moving against gravity. Muscle strength was measured according to the manual muscle test (MMT8) by rheumatologists (9). Cutaneous involvement included specific rashes (heliotrope rash, Gottron sign, and Gottron papules), non-specific rashes (V sign, shawl sign, holster sign, mechanic’s hands, and cutaneous ulcer), subcutaneous calcification, and subcutaneous edema. Cardiac involvement included abnormal electrocardiogram (ECG) and/or abnormal echocardiography (Echo), which could not be explained by ailments besides myositis. GI involvement primarily consisted of dysphagia and GI ulcers, which were confirmed by electronic endoscopic examination. Arthritis was recorded if the patients complained of joint pain and swelling. High-resolution computed tomography (HRCT) scans were employed to confirm the presence of ILD (10). Rapidly progressive ILD (RP-ILD) was defined as the presence of progressive dyspnea and worsening interstitial abnormalities detected by HRCT within 1 month from the onset of respiratory symptoms. Cancer-associated myositis was defined as cancer occurring within 3 years before or after disease onset.

### Laboratory data of anti-NXP2 positive myositis

Laboratory data consisted of a routine blood test, lymphocyte subsets, the spectrum of muscle enzymes (alanine aminotransferase-ALT, aspartate aminotransferase-AST, creatine kinase-CK, lactate dehydrogenase-LDH), albumin and pre-albumin, complement C3 and C4, C-reactive protein, serum ferritin, fibrinogen, D-dimer, anti-nuclear antibody (ANA) spectrum and myositis associated antibody (MAA). The titer of ANA ≥ 1:80 was considered positive (11).

### Detection of MSA antibody

MSA detection was conducted using the sample of the patient’s first admission to our department. We used the EUROLINE Autoimmune Inflammatory Myopathies Ag (IgG) test kit to complete MSA assay, according to the manufacturer’s protocol (order no. DL 1530-1,601-4G; EUROIMMUN). The positive control and the negative control sample were provided by the test kit. Then EUROBlotOne (EUROIMMUN) was used to detect the signal intensity. The positive MSA was defined as a result above 25.

### Treatment and follow-up of anti-NXP2 positive myositis

For the treatment of patients with anti-NXP2 positive myositis, the initial dose of glucocorticoids (GC) and disease-modifying anti-rheumatic drugs (DMARDs) were recorded. GC pulse therapy was defined as a dose above 125 mg daily (methylprednisolone equivalent) (12). Some patients received intravenous immunoglobulin (IVIG) as a combined therapy, which was also included in the study.

We conducted follow-up for all patients by telephone in December 2023. The content mainly concerned on the patients’ disease status, in particular, whether they had relapse, the time of the first relapse, and the dose of GC. The survival time was defined as the interval between symptom onset and death in patients who died or last follow-up date in patients who survived. The relapse was recorded as clinical, laboratory, or radiological deterioration during GC tapering or DMARDs adjustment, following a treatment-induced remission.

The disease activity was evaluated by clinicians based on an overall assessment. The high scores on the visual analog scale (VAS; score range, 0–10) indicated severe disease activity. Furthermore, disease activity was estimated based on the mean scores of two rheumatologists (L. L. and L. S.).

### Statistical analysis

SPSS (version 21.0; IBM Corp) was used for statistical analyses. The distribution of each continuous parameter was analyzed by Kolmogorov–Smirnov test. As for the statistical differences in different groups, t tests (normal distribution), Mann–Whitney U tests (nonnormal distribution), or chi-square test was calculated. For overall survival analysis, Kaplan–Meier curves were obtained for different groups, and log-rank test was performed. The statistical tests were two-sided, and the significance was defined as *p* < 0.05.

## Results

### Characteristics of patients with anti-NXP2 antibody

One hundred ten adult patients with anti-NXP2 antibody myositis were enrolled in the cohort, of whom 88 diagnosed with DM and 9 were classified as having DMSD. Of the remaining 13 patients, 6 patients were diagnosed with DMSD (cDMSD) clinically because of the lack of muscle biopsy, presenting with severe muscle weakness and strong positive anti-NXP2 antibody without any rashes. Seven patients with non-specific rashes and muscle weakness were also included in the cohort due to the strong positive anti-NXP2 antibody. Among the patients in the study, 11 patients had other connective tissue diseases, including 3 cases with rheumatoid arthritis, 3 cases with Sjogren’s disease, 3 cases with systemic sclerosis, 2 cases with ulcerative colitis, and 1 case with primary biliary cholangitis.

There were 63 (57.3%) female patients in the cohort. The average onset age was 42.47 ± 14.72 years old. The duration of the disease was 4 (1, 12) months, and 26 patients received initial treatment. A total of 107 patients experienced muscle weakness during the disease course, including 53 patients with distal limb weakness. In addition, 95 patients had cutaneous involvement, with the frequencies of heliotrope rash, Gottron sign, and Gottron papules being 66.4, 32.7, and 29.1%, respectively. In terms of other system involvement, 61 cases had cardiac involvement, including 49 cases with abnormal ECG and 22 cases with abnormal Echo. There were 75 patients who reported dysphagia, and 12 patients had GI ulcers, including one with cancer. Furthermore, 14 patients experienced arthritis, and 50 patients were diagnosed with ILD by HRCT, in which non-specific interstitial pneumonia was the most common pattern for 41 patients. No RP-ILD was found in the cohort.

We also compared the frequency of distal limb weakness in patients with different MSA DM, which was shown in Table 1. It was obvious that patients with anti-NXP2 positive myositis showed the highest percentage of distal limb weakness, followed by patients with anti-TIF1γ antibody and anti-Mi2 antibody. There existed significant difference between patients with and without anti-NXP2 antibody, which indicated that distal limb weakness might be a characteristic in anti-NXP2 positive myositis.

**Table 1:** The comparison of distal limb involvement frequency among DM patients with different MSA.

Clinical features	Anti-NXP2 *n* = 110	Non-anti-NXP2 *n* = 100	Anti-Mi2 *n* = 20	Anti-TIF1γ *n* = 20	Anti-SAE *n* = 20	Anti-MDA5 *n* = 20	MSA- *n* = 20	p*	p**
Age (y)	44.11 ± 14.76	51.28 ± 12.77	49.90 ± 15.39	52.65 ± 13.70	56.60 ± 9.51	52.15 ± 10.04	45.10 ± 12.64	<0.001	0.001
Gender F (*n*)	63	65	10	13	16	14	12	0.252	0.356
Neck (*n*)	87	46	13	12	5	4	12	<0.001	<0.001
Proximal (*n*)	101	73	15	16	13	14	15	<0.001	0.002
Upper (*n*)	94	58	12	15	12	9	10	<0.001	<0.001
Lower (*n*)	93	68	14	16	12	14	12	0.005	0.057
Distal (*n*)	53	23	6	7	4	2	4	<0.001	0.003
Upper (*n*)	51	22	6	7	4	2	3	<0.001	0.004
Lower (*n*)	48	18	4	7	2	2	3	<0.001	0.001

*p-value between patients with anti-NXP2 and non-anti-NXP2 antibody.

**p-value among the six subgroups.

### Comparison of clinical features and laboratory data in patients with anti-NXP2 positive myositis

Patients with anti-NXP2 positive myositis were stratified according to the presence or absence of distal limb weakness. The clinical features and laboratory data were analyzed to further clarify the differences between these two subgroups (Table 2).

**Table 2:** The clinical data between patients with and without distal limb involvement.

Patient characteristics	With (*n* = 53)	Without (*n* = 57)	*p*
Gender (F)	31 (58.5)	32 (56.1)	0.803
Onset age (y)	38.40 ± 13.36	46.26 ± 15.02	0.005*
Disease duration (m)	4 (1, 12)	4 (2, 12)	0.343
Initial treatment (1842327 %)	10 (18.9)	16 (28.1)	0.256
Myalgia (*n* %)	47 (88.7)	44 (77.2)	0.111
Muscle weakness (*n* %)	53 (100)	54 (94.7)	0.268
Severe muscle weakness (*n* %)	47 (88.7)	13 (22.8)	<0.001**
Neck (*n* %)	50 (94.3)	37 (64.9)	<0.001**
Proximal (*n* %)	53 (100)	48 (84.2)	0.008*
Upper (*n* %)	52 (98.1)	42 (73.7)	<0.001**
Lower (*n* %)	52 (98.1)	41 (71.9)	<0.001**
Distal			
Upper (*n* %)	51 (96.2)	0	<0.001**
Lower (*n* %)	48 (90.6)	0	<0.001**
Rash (*n* %)	45 (84.9)	50 (87.7)	0.667
Heliotrope rash (*n* %)	36 (67.9)	37 (64.9)	0.738
Gottron sign (*n* %)	17 (32.1)	19 (33.3)	0.888
Gottron papules (*n* %)	15 (28.3)	17 (29.8)	0.861
V sign (*n* %)	26 (49.1)	32 (56.1)	0.457
Shawl sign (*n* %)	23 (43.4)	21 (36.8)	0.483
Holster sign (*n* %)	7 (13.2)	9 (15.8)	0.701
Mechanic’s hands (*n* %)	6 (11.3)	6 (10.5)	0.894
Cutaneous ulcer (*n* %)	12 (22.6)	11 (19.3)	0.667
Subcutaneous calcification (*n* %)	5 (9.4)	9 (15.8)	0.318
Subcutaneous edema (*n* %)	34 (64.2)	20 (35.1)	0.002*
Rash VAS	3 (1, 3)	2 (1, 3)	0.481
Arthritis (*n* %)	5 (9.4)	9 (15.8)	0.318
GI involvement			
Dysphagia (*n* %)	42 (79.2)	33 (57.9)	0.016*
GI ulcer (*n* %)	9 (17.0)	3 (5.3)	0.049*
ILD (*n* %)	26 (49.1)	24 (42.1)	0.464
Cardiac involvement			
Abnormal ECG (*n* %)	31 (58.5)	18 (31.6)	0.005*
Abnormal Echo (*n* %)	10 (18.9)	12 (21.1)	0.775
Disease activity VAS	6 (5, 7)	4 (3, 5)	<0.001**
WBC (10^9^/L)	8.83 ± 4.28	8.28 ± 3.60	0.460
Neu (10^9^/L)	7.17 ± 4.16	6.49 ± 3.46	0.357
Lym (10^9^/L)	1.00 ± 0.57	1.14 ± 0.53	0.213
CD4 (/uL)	462 ± 277	568 ± 316	0.074
CD8 (/uL)	239 ± 189	273 ± 174	0.343
CD4/CD8	2.12 (1.25, 4.15)	1.83 (1.33, 3.71)	0.937
NK (/uL)	45 (21,69)	47 (27,105)	0.103
B (/uL)	203 (125,395)	228 (87, 453)	0.754
Neu/Lym	7.07 (3.77, 11.58)	5.99 (3.13,9.16)	0.111
Hb (g/L)	122 ± 16	122 ± 15	0.944
PLT (10^9^/L)	181.32 ± 74.06	202.37 ± 68.50	0.124
Muscle enzyme profile			
ALT (U/L)	55 (31,150)	49 (23,81)	0.068
AST (U/L)	90 (47, 257)	37 (21, 123)	<0.001**
CK (U/L)	1,419 (219, 4,210)	233 (70,1,401)	0.001*
CKmax (U/L)	5,033 (2,271, 10,888)	1828 (288,3,498)	<0.001**
LDH (U/L)	491 (339, 744)	331 (249, 492)	0.001*
Alb (g/L)	34 (31, 37)	37 (34, 39)	0.004*
ProAlb (mg/L)	188 ± 84	195 ± 104	0.702
IgG (mg/dL)	1,201 ± 439	1,295 ± 639	0.381
IgA (mg/dL)	183 (131,258)	197 (152,277)	0.345
IgM (mg/dL)	115 (76, 158)	103 (79,151)	0.892
C3 (mg/dL)	78.80 ± 17.79	84.14 ± 16.24	0.106
C4 (mg/dL)	19.51 ± 5.11	18.74 ± 5.37	0.449
CRP (mg/dL)	0.61 (0.36,1.02)	0.44 (0.20,1.04)	0.227
Serum ferritin (*n*g/mL)	506 (144,897)	168 (56, 418)	0.001*
Fib (g/L)	3.32 ± 1.15	3.77 ± 1.36	0.078
D-dimer (mg/L)	1.56 ± 1.02	1.19 ± 0.79	0.051
ANA	25 (47.2)	26 (45.6)	0.571
MAA	7 (13.2)	13 (22.8)	0.192

*p < 0.05, **p < 0.001.

GI gastrointestinal, ILD interstitial lung disease, VAS visual analog scale.

Patients with distal limb weakness were younger at disease onset. The average age of onset in the distal limb weakness group was 38.40 ± 13.36 years old, while in the other group, it was 46.26 ± 15.02 years old with a statistically significant difference (*p* = 0.005). The incidence of severe muscle weakness was significantly higher in patients with distal limb weakness (88.7% vs. 22.8%, *p* < 0.001). The proportion of proximal limb and neck muscle involvement was also higher in this group. In addition, patients with distal limb weakness had a higher frequency of subcutaneous edema (64.2% vs. 35.1%, *p* = 0.002), whereas there was no significant difference in the proportion of other cutaneous involvement and the VAS of rashes. Meanwhile, the patients with distal limb weakness seemed to be more likely to develop GI involvement, with higher incidences of dysphagia (79.2% vs. 57.9%, *p* = 0.016) and GI ulcers (17.0% vs. 5.3%, *p* = 0.049), respectively. The frequency of ILD occurrence was equivalent in both groups. The distal limb weakness group had a relatively higher incidence of abnormal ECG. Cardiac arrhythmia was the most common, including 23 patients with sinus tachycardia, 4 with ventricular premature beats, and 3 with atrial premature beats.

As for the laboratory data, patients in the distal limb involvement group had higher levels of muscle enzyme profiles, including AST, CK, and LDH, with significant differences. Furthermore, the maximum level of CK during the disease course was significantly higher than that of the control group [5,033 (2,271, 10,888) U/L vs. 1828 (288, 3,498) U/L], with *p* value of less than 0.001. In this group, patients had relatively lower levels of albumin [(34.00 (30.75, 37.00)g/L vs. 37.00 (33.95, 39.35)g/L, *p* = 0.004)]. However, their serum ferritin levels were significantly elevated and the difference was significant [505.55 (143.65, 897.45) ng/mL vs. 168.30 (55.90, 418.00)ng/mL, *p* = 0.001]. In summary, VAS results showed that patients with distal limb weakness had higher disease activity compared to patients without distal limb weakness [6 (5, 7) vs. 4 (3, 5), *p* < 0.001].

### Treatment and follow-up of patients with distal limb weakness

There were 32.1% of patients with distal limb weakness receiving GC pulse therapy during the disease course, which was significantly higher than that in patients without distal limb weakness (Table 3). Fifty patients received concurrent DMARDs therapy. Among the remaining 3 patients who did not receive DMARDs, one had cancer, one had hepatitis B virus infection, and the third did not take DMARDs for unknown reasons. Eighteen patients received biological or targeted synthetic DMARDs (b/tsDMARDs), including Tocilizumab (10 cases), JAK inhibitors (9 cases), and Rituximab (2 cases). Among them, 3 patients received two kinds of agents sequentially. In the distal limb weakness group, 36 patients (67.9%) received IVIG as part of their treatment. In the other group, only 14 patients (24.6%) received IVIG, which was significantly lower.

**Table 3:** The treatment and follow-up between patients with and without distal limb involvement.

Outcomes	With (*n* = 53)	Without (*n* = 57)	*p*
Treatment^a^			
GC (*n*)	53 (100)	57 (100)	1.000
GC pulse (*n* %)	17 (32.1)	4 (7.0)	0.001*
DMARDs (*n* %)	50 (94.3)	52 (91.2)	0.794
b/tsDMARDs (*n* %)	18 (34.0)	8 (14.0)	0.014*
IVIG (*n* %)	36 (67.9)	14 (24.6)	<0.001**
Replase^b^ (*n* %)	32 (69.6)	24 (52.2)	0.087
GC dose (mg/d) at the first relapse	10.20 ± 8.08	6.30 ± 6.61	0.056
In the 1st year (*n* %)	21 (45.7)	10 (21.7)	0.015*
In the 2nd year (*n* %)	7 (15.2)	5 (10.9)	0.536
Death^c^ (*n* %)	9 (21.4)	9 (20.0)	0.869
Survival time (m)	20.00 ± 21.32	24.33 ± 20.29	0.665
In the 1st year (*n* %)	5 (11.9)	1 (2.2)	0.175
In the 2nd year (*n* %)	2 (4.8)	4 (8.9)	0.737
Infection (*n* %)	5 (11.9)	2 (4.4)	0.377
Cancer (*n* %)	2 (4.8)	4 (8.9)	0.737
Disease progression (*n* %)	1 (2.4)	2 (4.4)	1.000
Others (*n* %)	1 (2.4)	1 (2.2)	1.000
Cancer^d^ (*n* %)	3 (7.1)	4 (8.9)	1.000

*p < 0.05, **p < 0.001.

^a^Data available for 110 patients. ^b^Data available for 92 patients with 46 in distal involvement group. ^c,d^Data available for 87 patients with 42 in distal involvement group.

By December 2023, 32 patients in the distal limb weakness group experienced relapse during the disease course. Among these, 21 cases relapsed in the first year, and 7 cases relapsed in the second year. In the other group, 24 patients experienced relapse during the disease course, with 10 and 5 patients relapsing in the first and second year, respectively. Although the overall relapse rate did not differ significantly between the two groups, the distal limb weakness group had a higher relapse rate in the first year (*p* = 0.015). The mean daily GC dose (prednisone equivalent) at disease relapse was 10.20 ± 8.08 mg in the distal limb weakness group, which was higher than that in the other group (6.30 ± 6.61 mg); however, the difference was not statistically significant (*p* = 0.056).

**Figure 1:**
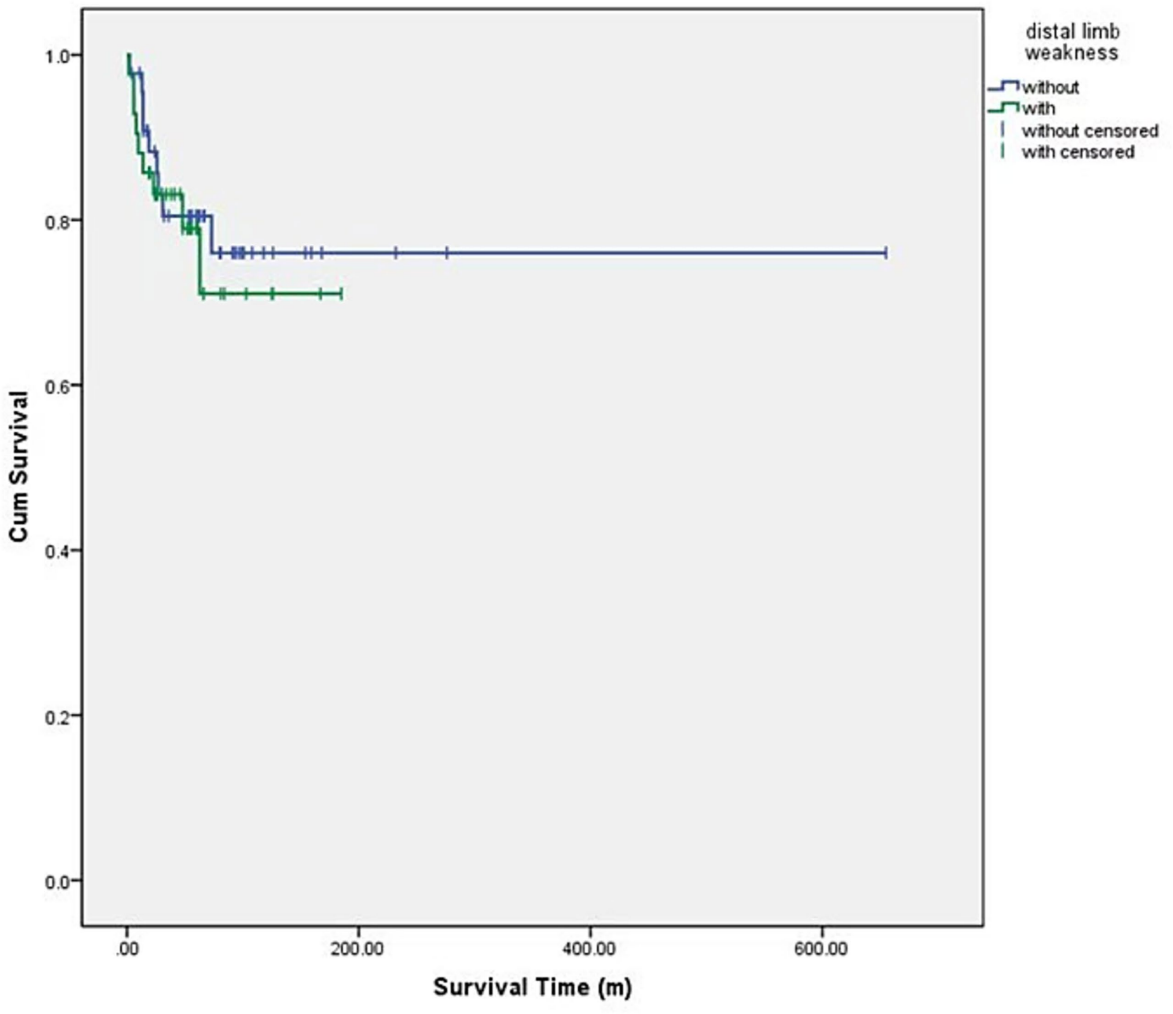
The overall survival time between patients with and without distal limb weakness.

We next analyzed the overall survival status of patients with distal limb weakness. Eleven patients were lost to follow-up due to invalid contact information. Among the remaining 42 patients, 9 patients died. The causes of death included infection (*n* = 5), tumor (*n* = 2), disease progression (*n* = 1), and GI perforation (*n* = 1). All patients who died of infection were in a state of active disease. The mean survival time of these 9 patients was 20.00 ± 21.32 months. Compared with the other group, there was no difference in overall survival rate, as shown in Figure 1 (*p* = 0.668). However, the distal limb weakness group appeared to have an increase first-year mortality rate and a higher risk death due to infection.

## Discussion

In our cohort, nearly half of adult patients with anti-NXP2 positive myositis experienced distal limb weakness. Our study elucidated the clinical characteristics and prognosis of this subgroup based on a relatively large cohort. These patients exhibited severe disease activity and a relatively high frequency of relapse.

Within the IIM spectrum, patients with DM typically present with proximal limb weakness. However, nearly half of adult patients with anti-NXP2 positive myositis in our cohort developed distal limb weakness, a finding more frequent than that reported in previous studies (3, 13). Moreover, this phenomenon was more common in adult patients than in juvenile anti-NXP2 positive DM (14). Our results indicated that distal limb weakness in patients with anti-NXP2 positive myositis was often associated with severe muscle weakness and high disease activity, as evidenced by significantly elevated peak CK levels and a higher proportion of severe muscle weakness. Previous studies have suggested that the severity of muscle weakness correlated with muscle biopsy characteristics, particularly in cases with ischemia and infarction (4). Compared with other MSA positive DM, micro-infarction was a characteristic pathological feature of anti-NXP2 positive myositis (15, 16). Therefore, further research on muscle pathology in these patients would provide valuable insight into disease status.

The incidence of specific and non-specific rashes in patients with distal limb weakness was comparable to that in other patients, except for subcutaneous edema. Subcutaneous edema was the second most common cutaneous manifestation in the distal limb weakness group, though its incidence was lower than that of heliotrope rash. Subcutaneous edema is another well-known typical feature of anti-NXP2 positive myositis (3, 17). Studies have suggested that the occurrence of subcutaneous edema may be related to muscle fiber necrosis (3). Thus, distal limb weakness and subcutaneous edema were more prominent in anti-NXP2 positive myositis than in other DM subtypes, which may be attributable to the specific pathological state of the muscles.

Distal limb weakness was not only associated with isolated muscle damage, but also with multiple systems involvement. In this study, GI involvement was particularly evident in this subgroup, especially dysphagia, which was consistent with previous study (18). GI vasculitis, manifesting as ulcers, perforations, and bleeding, has recently garnered increasing attention due to its poor prognosis. Given the pathological findings of thrombosis and necrosis in GI biopsy, this may also be related to the underlying pathological characteristics of anti-NXP2-positive myositis (7, 19, 20). Additionally, the proportion of ECG abnormalities, particularly cardiac arrhythmia, was relatively high among patients with distal limb weakness. The specific mechanisms underlying these associations remain unclear and warrant further investigation (21).

Patients with distal limb weakness required more aggressive treatment, including GC pulse, b/tsDMARDs, and IVIG, as this subgroup exhibited higher disease activity. According to a previous study, the overall relapse rate in patients with anti-NXP2 positive myositis was 68.75%, which was consistent with our findings (22). However, even with active treatment, we found that the relapse rate among patients with distal limb weakness, particularly within the first year, was significantly increased. In addition, the GC dose at the time of relapse was also higher in this group. Therefore, vigilance is warranted during treatment to modify therapeutic strategies. The adjustment of GC and DMARDs appeared to require a slower and more gradual tapering process. Although previous studies have shown that patients with anti-NXP2 positive myositis had a higher likelihood of medication cessation, the goal of early disease treatment should focus on disease control and relapse reduction (23). Furthermore, IVIG may represent a common and appropriate adjunctive treatment option for these patients. Recent studies have provided satisfactory recommendation for IVIG in severe IIM (24, 25). Although our study suggested no significant difference in survival rates between patients with and without distal limb weakness, it was worth noting that the first-year mortality rate in this group appeared to be higher, with infections being the primary cause of death, which was consistent with previous studies (26). Therefore, close monitoring for the infections during treatment remains essential.

Our research has some limitations. First, this was a retrospective study in which clinical data were collected from medical records and follow-up data were based on patient self-reports. For some initially abnormal laboratory results or tests, no follow-up information on treatment response was available. Therefore, we used qualitative analysis to minimize potential bias. Second, our study was conducted at a single center with a long enrollment period. Treatment options, particularly for DMARDs, have evolved over time, which may lead to inconsistent prognoses across different stages. For example, patients enrolled recently presented with severe conditions and received various kinds of DMARDs, potentially introducing selection bias. Finally, antibody detection was performed using immunoblotting, a method that can yield false positive or false negative results. However, the EUROLINE test kits are now commonly used in clinical practice. Several studies have demonstrated the accuracy of immunoblotting due to its high specificity (27). Accordingly, multi-center prospective studies conducted within a consistent time frame are urgently needed to obtain more robust conclusions and inform clinical practice.

## Conclusion

Distal limb weakness is a common characteristic in patients with anti-NXP2 positive myositis. They typically present at young onset ages and exhibit severe disease activities, often involving multiple systems. Moreover, despite initial aggressive treatment, their relapse rate remains relatively high.

## Data Availability

The original contributions presented in the study are included in the article/supplementary material, further inquiries can be directed to the corresponding author.
